# Decellularizing corneal stroma using N_2_ gas

**Published:** 2008-05-14

**Authors:** Shiro Amano, Naoki Shimomura, Seiichi Yokoo, Kaoru Araki-Sasaki, Satoru Yamagami

**Affiliations:** 1 Department of Ophthalmology, University of Tokyo School of Medicine, Tokyo, Japan; 2 Department of Corneal Tissue Regeneration, University of Tokyo Graduate School of Medicine, Tokyo, Japan; 3 Ideta Eye Hospital, Kumamoto, Japan

## Abstract

**Purpose:**

To examine the efficacy of a novel method of decellularizing porcine corneal stroma using N_2_ gas from liquid N_2_ and the feasibility of using decellularized porcine corneal stroma in a corneal transplantation model in rabbits.

**Methods:**

Porcine corneas were placed in a tube, and N_2_ gas from liquid N_2_ was poured into the tube to freeze the corneas and make the inside of the tube hypoxic. After fastening the cap firmly, the tube was kept at room temperature for seven days, and the porcine corneas were examined histologically. A porcine corneal stromal disk treated with the aforementioned method was inserted into a pocket of rabbit corneal stroma and observed for six months.

**Results:**

Hoechst 33342 and hematoxylin and eosin staining both showed few cellular nuclei in the porcine corneal stroma incubated in N_2_ gas for one week. A terminal deoxynucleotidyl transferase-mediated dUTP nick end-labeling (TUNEL) assay showed many positively stained nuclei in the porcine corneal stroma incubated in N_2_ gas for three days. The porcine corneal stroma that was decellularized and transplanted into a rabbit corneal stromal pocket remained clear for six months after transplantation.

**Conclusions:**

This method using N_2_ gas decellularizes corneal stroma without reducing corneal transparency.

## Introduction

While corneal transplantation is very useful, a grave shortage of corneas exists worldwide. To solve this problem, several researchers have tried to tissue-engineer corneas using cultured cells and the extracellular matrix [1-5]. Although human corneal stroma is the most appropriate source of corneal stroma for tissue-engineering the cornea, its supply is limited. Natural and synthetic polymers have been used to produce scaffolds for tissue-engineered corneas, but they are not usable clinically [6,7]. Consequently, the corneal stroma of animals may be a good source for tissue-engineering corneas.

The pig has been investigated extensively as a candidate donor for xenografting because pigs are bred and consumed for food on a large scale and are considered appropriate for use as donors both commercially and ethically. Previously, we found that the α-gal epitope, which induces hyperacute rejection, is almost absent in the porcine cornea except for several keratocytes in the anterior-most part, suggesting that the porcine cornea has clinical potential [8]. However, when xenotransplantation using porcine organs or tissues is performed, cross-species transmission of porcine pathogens is a major concern. Therefore, decellularized porcine corneal stroma may warrant evaluation because this process would reduce the possibility of pathogen transmission.

Although a stromal transplant can be used only when the recipient’s corneal endothelium is intact, one of the requirements of a decellularized corneal stroma is that it maintains its clarity. While various methods of tissue decellularization have been reported including snap freezing, mechanical force, ionic and nonionic detergents, ethylenediaminetetraacetic acid(EDTA), and enzymatic treatment [9], their ability to decellularize the corneal stroma without reducing the transparency of the cornea has not been evaluated. Therefore, we examined the efficacy of a novel method of decellularizing porcine corneal stroma and the feasibility of using them in a corneal transplantation model in rabbits.

## Methods

### Decellularization of porcine corneas

Twelve fresh pig eyes were obtained from a slaughterhouse, and whole corneas were excised from these eyes. Four porcine corneas were placed in a 50 ml tube (Corning, Corning, NY), and N_2_ gas from liquid N_2_ was poured into the tube to freeze the corneas and make the inside of the tube hypoxic (N_2_ group). As a control group, four fresh porcine corneas were placed in a tube with air and kept under normoxic conditions (control group 1). As another control group, four fresh porcine corneas were frozen with liquid N_2_ outside the 50 ml tube and placed in a 50 ml tube with air (control group 2). After fastening the caps firmly, the tubes were kept at room temperature. Porcine corneas were examined histologically before and then three, five, and seven days after the treatment.

### Histological examination

The porcine corneas were fixed in a buffered 8% paraformaldehyde, embedded in paraffin, cut into 4 µm thick sections, and placed on Matsunami adhesive silane (MAS)-coated microscope slides (Matsunami Glass, Tokyo, Japan). The sections were then stained with hematoxylin and eosin or Hoechst 33342. The slides were observed under a light or fluorescence microscope. The numbers of corneal stromal cells were counted in a 500 µm^2^ area in the central corneal stroma. The numbers of Hoechst 33342-positive cellular nuclei in each group on day 0, 3, 5, and 7 were compared using repeated measurements analysis of variance (ANOVA) and the Bonferroni post hoc comparison.

### Apoptosis detection

To determine the mechanism of decellularization in porcine corneas, apoptosis was measured using terminal deoxynucleotidyl transferase-mediated nick end labeling (TUNEL) staining using the ApopTag^®^ in situ apoptosis detection kit (Intergen, Purchase, NY). We followed the manufacturer’s protocol to detect apoptosis in cells on 4 µm thick slices of corneal stroma. Cellular nuclei were stained with propidium iodide, and apoptotic cells were observed under a phase-contrast light microscope.

### Transplantation of a decellularized porcine cornea into rabbit corneal stromal pockets

The protocol used for these animal experiments adhered to the Association for Research in Vision and Ophthalmology (ARVO) statement for the use of animals in ophthalmic and vision research. The central cornea was ablated with a microkeratome (MK-2000; Nidek, Aichi, Japan) to obtain porcine corneal stromal disks using a 7.0 mm suction ring and a 160 µm microkeratome head. By applying the microkeratome to the center of the porcine cornea several times, three to four disks were produced from different layers of a porcine cornea. Residual corneal thickness was measured with an ultrasound pachymeter (SP-2000; Tomey, Nagoya, Japan), and the thickness of the resulting corneal disks was calculated. The corneal stromal disks had diameters of approximately 5 mm and thicknesses of 100–200 μm. The corneal stromal disks from the middle layer of the porcine cornea were placed in a 50 ml tube filled with N_2_ gas from liquid N_2_ to keep the cornea stromal disks hypoxic. After fastening the caps firmly, the tubes were kept in a refrigerator for seven days. Four New Zealand white rabbits were anesthetized with both intramuscular ketamine hydrochloride (20 mg/kg) and xylazine (20 mg/kg) as well as with topical 0.4% oxybuprocaine hydrochloride. A circular half-thickness keratotomy with a 6 mm diameter was performed at the central cornea of the right eyes of the rabbits using a Hessburg–Barron trephine (Katena Products, Denville, NJ). The lamella was dissected with a crescent knife (Mani, Tochigi, Japan) to make an intrastromal pocket. A 5 mm decellularized corneal stromal disk was inserted into the pocket, and the incision was closed with two to four 10–0 nylon sutures. Anterior eye segments were observed and photographed with a photo-slit camera (Genesis; Kowa, Tokyo, Japan) one, three, and six months after surgery. Six months after surgery, the rabbits were killed with an overdose of intravenous pentobarbital under deep anesthesia with intramuscular ketamine hydrochloride (25 mg/kg) and xylazine (10 mg/kg). The eyes were enucleated and examined histologically.

## Results

### Histological examination

**Figure 1:**
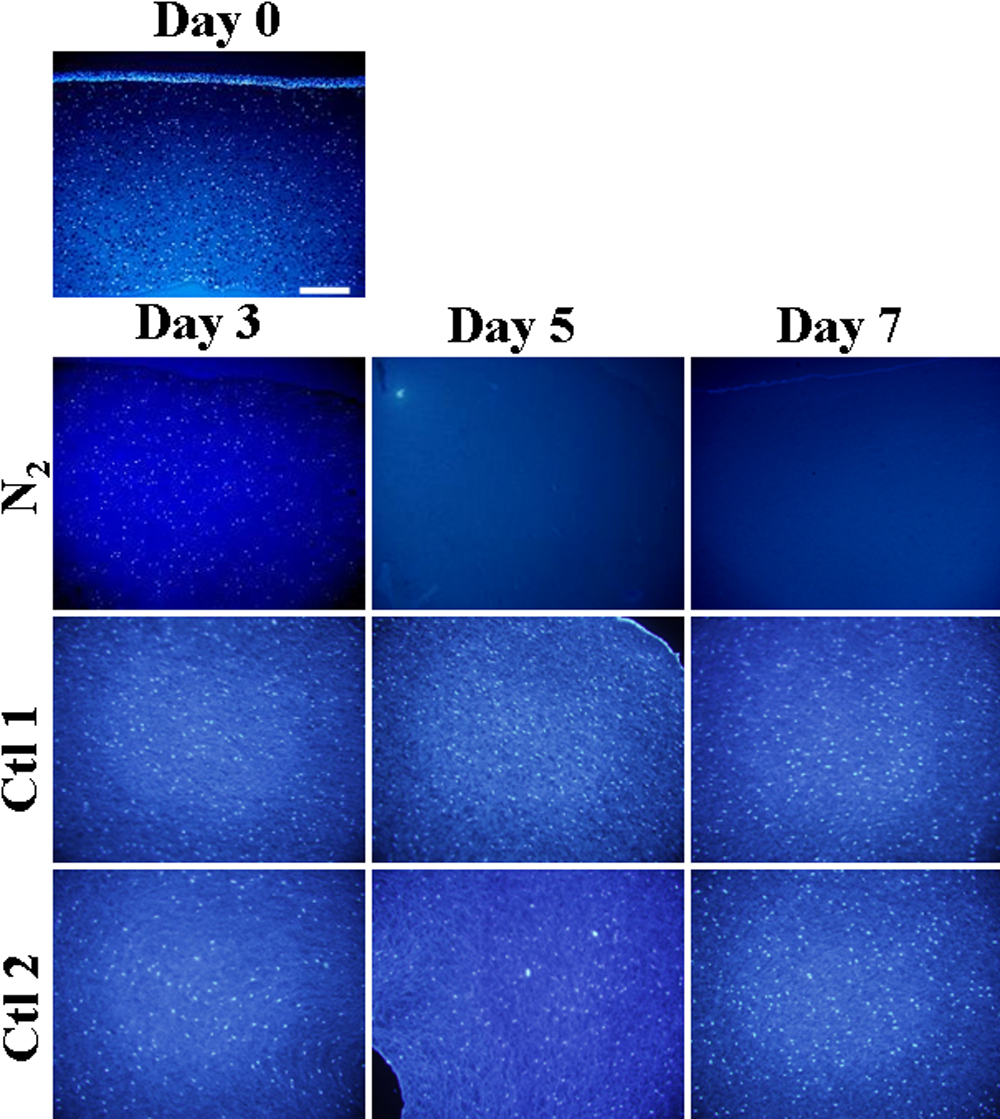
Hoechst 33342 staining of porcine corneas. Porcine corneas were incubated for one week in N_2_ gas (N_2_ group), air without freezing (control 1 group [Ctl 1]), or air after freezing (control 2 group [Ctl 2]). Fewer cellular nuclei were present in the porcine cornea incubated in N_2_ gas for three days than in the two control groups. After five or seven days, few cellular nuclei were observed in the porcine corneal stroma incubated in N_2_ gas while many keratocyte nuclei were observed throughout the corneal stroma in the control groups that were kept in air for five or seven days with or without freezing. Positively stained epithelial cells were observed at day 0. All small figures are at the same magnification, and the bar is 100 µm.

**Figure 2:**
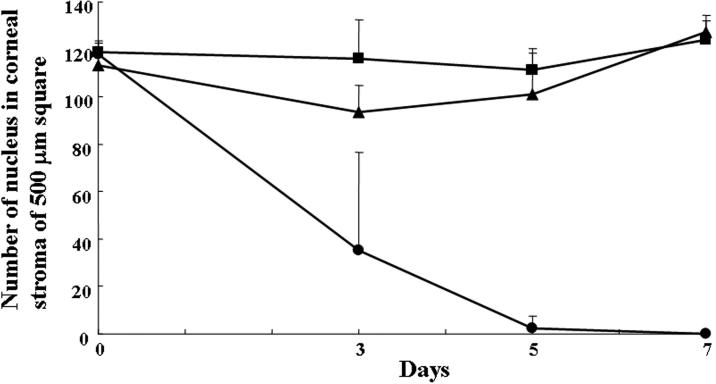
Temporal changes in the number of cellular nuclei in the N_2_ group and the control groups. The temporal changes in the number of cellular nuclei stained with Hoechst 33342 in a 500 μm^2^ area of the central corneal stroma in the N_2_ group (closed circles), control group 1 (closed squares), and control group 2 (closed triangles) is shown in this chart. Significantly fewer stained cellular nuclei were observed in the corneal stroma of the N_2_ group than in the control groups on day 3 (p=0.0054), 5 (p<0.0001), and 7 (p<0.0001).

Hoechst 33342 staining showed fewer cellular nuclei in the porcine corneal stroma incubated in N_2_ gas for three days than in the two control groups (Figure 1). In the porcine corneal stroma incubated in N_2_ gas for five or seven days, no cellular nuclei were observed. In contrast, many keratocyte nuclei were observed throughout the corneal stroma in the control groups that were kept in air for seven days with or without freezing with liquid N_2_ gas. The time-dependent decrease in the numbers of cellular nuclei stained with Hoechst 33342 in a 500 μm^2^ area of the central cornea stroma for each group is shown in Figure 2. Significantly fewer stained cellular nuclei were in the corneal stroma in the N_2_ group than in the control groups on day 3 (p=0.0054), 5 (p<0.0001), and 7 (p<0.0001).

**Figure 3:**
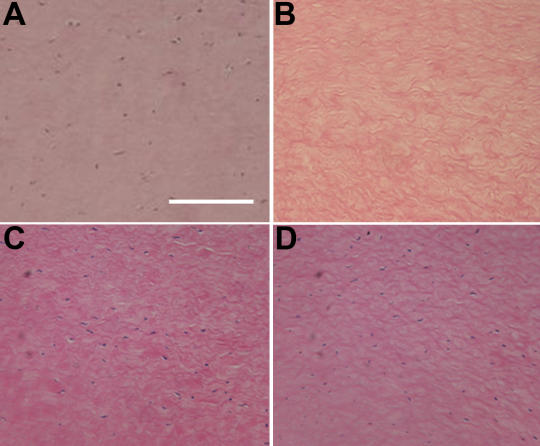
Hematoxylin and eosin staining of porcine corneal stroma. The porcine cornea stroma were stained before treatment (**A**) as well as after incubating for one week in N_2_ gas (**B**), in air without freezing (**C**), or in air after freezing (**D**). Hematoxylin and eosin staining showed few cellular nuclei in the corneal stroma of the N_2_ group on day 7 whereas many nuclei were observed in the corneal stroma of control groups 1 and 2 on day 7. All figures are at the same magnification, and the bar is 100 µm.

Hematoxylin and eosin staining showed few cellular nuclei in the corneal stroma of the N_2_ group at day 7 whereas many nuclei were observed in the corneal stroma of control groups 1 and 2 at day 7 (Figure 3).

**Figure 4:**
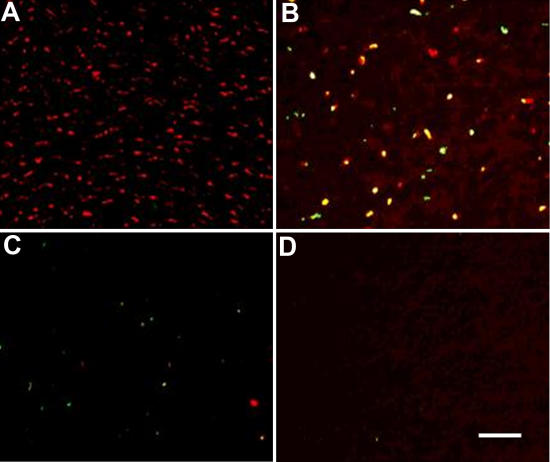
The results of the TUNEL assay of porcine corneal stroma. The TUNEL assay before treatment (**A**) and after incubation in N_2_ gas for three (**B**), five (**C**), and seven (**D**) days is shown. Cellular nuclei were stained with propidium iodide. Many positively stained nuclei (yellow dots in **C**) were observed at day 3 whereas no staining was recognized in the porcine corneas before treatment or after N_2_ incubation for five or seven days. All figures are at the same magnification, and the bar is 100 µm.

The TUNEL assay showed many positively stained nuclei in the porcine corneal stroma incubated in N_2_ gas for three days whereas the assay showed almost no staining in the porcine corneal stroma before treatment or after incubation in N_2_ gas for five or seven days (Figure 4).

### Transplantation of a decellularized porcine cornea into rabbit corneal stromal pockets

**Figure 5:**
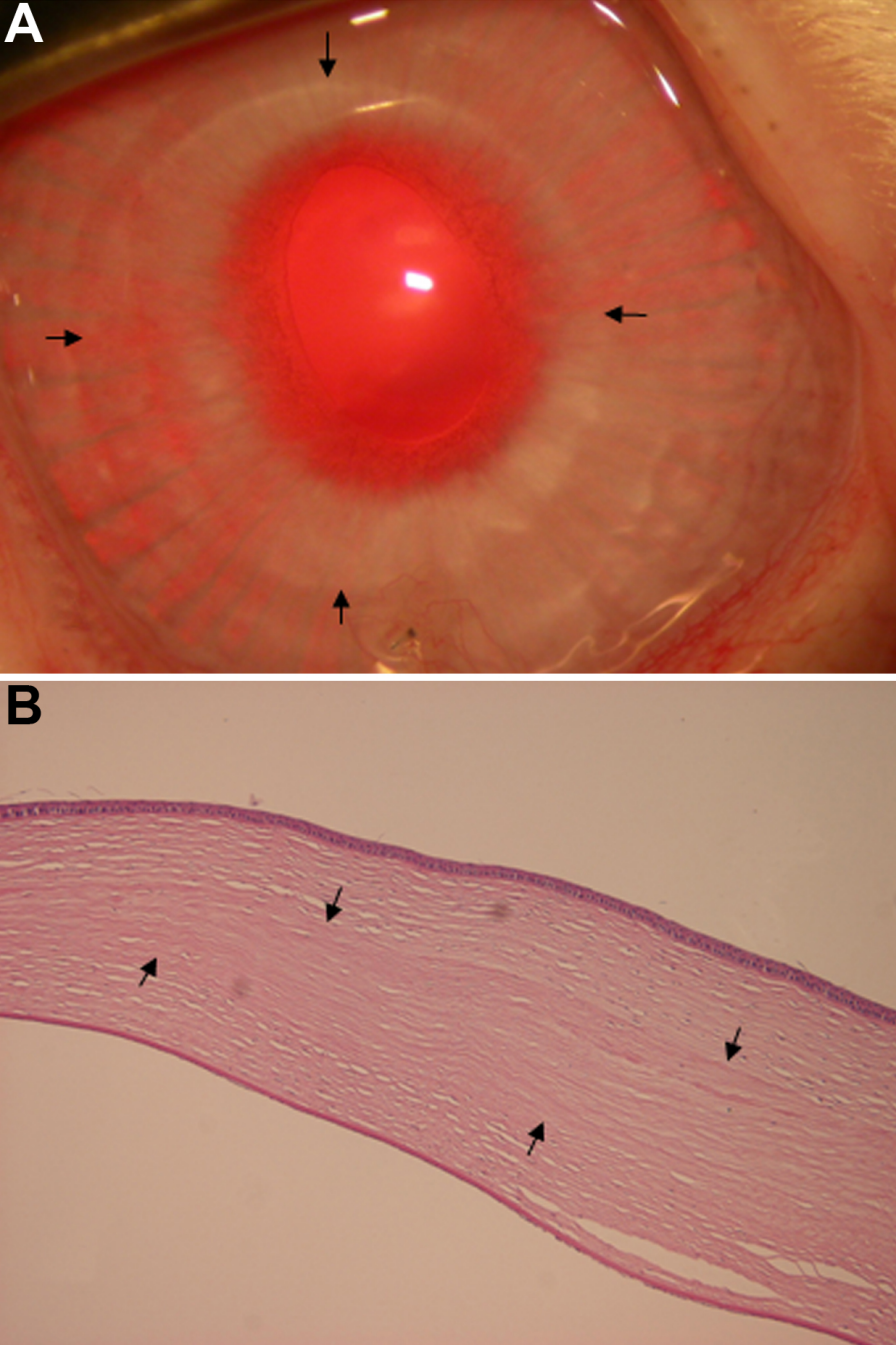
Anterior rabbit eye segments and histology six months after the transplantation of the decellularized porcine cornea. **A**: The transplanted porcine cornea (within the arrows) remained transparent for six months. **B**: The transplanted porcine cornea could be recognized in the rabbit cornea (arrows), and few keratocytes infiltrated the porcine cornea.

Three porcine corneal stroma disks that were incubated in N_2_ gas for one week were transplanted into rabbit corneal stromal pockets and remained clear for six months after transplantation (Figure 5A). No rejection or infection in or around the transplanted porcine corneas occurred. Histological examination six months after surgery showed that the transplanted porcine corneas could be recognized in the rabbit corneas and that few keratocytes infiltrated the porcine cornea (Figure 5B).

## Discussion

This study demonstrated that our method of decellularization using N_2_ gas can decellularize the porcine cornea entirely without reducing its transparency. Since the existing methods of decellularization such as mechanical force, ionic and nonionic detergents, EDTA, and enzymatic treatment involve extreme conditions, these methods are more likely to affect the transparency of the cornea. In contrast, our method uses relatively mild conditions and can maintain corneal transparency. Therefore, our method is likely to be superior to the other methods of decellularization because transparency is an essential property of the cornea.

The results of the TUNEL assay indicated that freezing and incubating in N_2_ gas induces keratocytes to undergo apoptosis within one week. In contrast, freezing and subsequent incubating in air did not decellularize the porcine corneal stroma in this study, although previous studies reported that a snap freezing method could decellularize ligaments and nerves [9]. Differences in the tissues and methods may account for this disparity. In our experiments, freezing alone did not decellularize the corneal stroma. The effect of hypoxia without freezing on the corneal stroma should be examined in a future study.

Histologic evaluation revealed that few keratocytes entered the decellularized porcine corneal stroma six months after they were transplanted into the rabbit corneal pocket. Furthermore, corneal transparency was maintained throughout the six months after transplantation. These findings suggest that corneal stromal transparency can be maintained for at least six months without keratocytes in the corneal stroma. However, because keratocytes may play some role in the maintenance and metabolism of the corneal stroma, the transparency of the transplanted decellularized porcine cornea must be observed for a longer period.

In conclusion, our method of decellularization using N_2_ gas can decellularize the porcine cornea entirely, and the decellularized cornea that was transplanted into the rabbit corneal stroma remained transparent for six months. This method may be practical for decellularizing the corneal stroma for clinical use.
